# Research Progress of Gas Sensing Performance of 2D Hexagonal WO_3_

**DOI:** 10.3389/fchem.2021.786607

**Published:** 2021-12-06

**Authors:** Yueqi Li, Qin Zhou, Shoubing Ding, Zhimin Wu

**Affiliations:** Chongqing Key Laboratory of Photoelectric Functional Materials, College of Physics and Electronic Engineering, Chongqing Normal University, Chongqing, China

**Keywords:** 2D, hexagonal WO_3_, gas sensing, oxygen vacancy, metal oxide semiconductor

## Abstract

Metal oxide semiconductor gas sensing materials have attracted great research interest in the gas sensor field due to their outstanding physical and chemical properties, low cost, and easy preparation. Among them, two-dimensional hexagonal tungsten trioxide (2D h-WO_3_) is especially interesting because of its high sensitivity and selectivity to some gases. We firstly introduce the characteristics of 2D h-WO_3_ gas sensing materials and discuss the effects of microstructure, oxygen vacancy, and doping modification on the gas sensing properties of 2D h-WO_3_ mainly. Finally, we explore the application of 2D h-WO_3_ gas sensing materials and propose some research directions.

## Introduction

As a critical component of the intelligent detection system, the gas sensor (Lee et al., 2018) has been widely used in environmental monitoring (Ji et al., 2019a), respiratory analysis, explosive gases, and automobile exhaust detection. Based on different working mechanisms, the developed gas sensors include semiconductor gas sensors (Morrison, 1987a; Zhang et al., 2021), polymer gas sensors (Zee and Judy, 2001), and electrochemical gas sensors (Tierney and Kim, 1993). Among them, the semiconductor gas sensors can also be divided into resistive and non-resistive types, while the resistive semiconductor gas sensors have advantages of high sensitivity and easy preparation (Seiyama et al., 1962). Meanwhile, compared with carbon and other organic gas sensing materials, the resistive metal oxide gas sensors (Nazemi et al., 2019) have become the research hotspot due to their high responsivity (Demarne and Grisel, 1988) and excellent selectivity (Morrison, 1987b). As a highly sensitive metal oxide gas sensing material, tungsten trioxide (WO_3_) has attracted extensive attention because of its unique physical and chemical properties (Salje and Viswanathan, 1975), and its applications in photocatalysis (Dong et al., 2017) and electrochromic (Adhikari and Sarkar, 2014).

**FIGURE 1:**
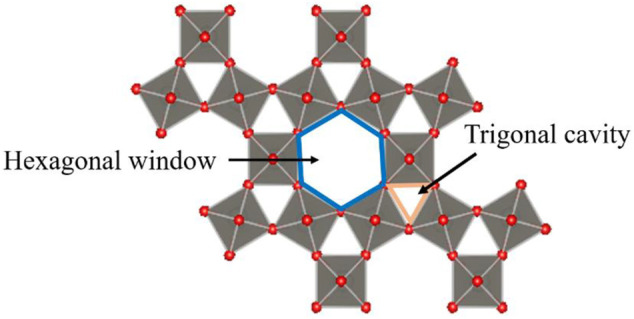
The hexagonal window and trigonal cavity of the hexagonal WO_3_(001) surface (Balaji et al., 2009).

WO_3_ is a typical metal oxide semiconductor with various phase transition structures, while different phases can induce different gas sensitivity. The stable structures at room temperature are m-WO_3_ and h-WO_3_. In recent years, as the most stable structure, m-WO_3_ has attracted much attention (Hübner et al., 2010; Oison et al., 2011), but bulk m-WO_3_ gas sensors are not sensitive to some gases at 25°C–500°C, such as CO (Ahsan et al., 2012) and H_2_S (Szilágyi et al., 2010). Therefore, it is urgent to improve the gas sensitivity of WO_3_ at room temperature effectively. Xu et al. (2008) found that the sensitivity of h-WO_3_ almost linearly increases with CO concentration at room temperature. Szilágyi et al. (2010) found that h-WO_3_ becomes more sensitive than m-WO_3_ compared to m-WO_3_ when the concentration of H_2_S is 10 ppm. Meanwhile, the large hexagonal and trigonal tunnel structures of h-WO_3_ result in it having a high specific surface area (as shown in Figure 1) (Balaji et al., 2009), indicating that h-WO_3_ is an excellent candidate material for gas sensors.

To effectively improve the gas sensitivity of the material, various preparation methods have been used to prepare WO_3_ nanomaterials on various dimensions (0D, 1D, 2D, and 3D) (Qin et al., 2010; Zhang et al., 2010; D'Arienzo et al., 2014). Among them, 2D nanomaterials are widely used because of their high specific surface area and abundant oxygen vacancies (Yang et al., 2016; Liu et al., 2017; Yang et al., 2017). The unique characteristics of 2D WO_3_ nanostructure compared with the bulk material include (1) higher specific surface area, which will provide more interaction area between tested gases and gas sensor surface molecules; (2) quantum confinement effects, due to the inherently small size of nanostructure materials, that can significantly affect charge transport, electronic band structure, and optical properties (Zheng et al., 2011). Based on this, we mainly review the effects of microstructure, oxygen vacancy, and doping modification on the gas sensing performance of 2D h-WO_3_ and explore the application prospect of the 2D h-WO_3_ gas sensor.

## Characteristics of 2D h-WO_3_ Gas Sensing Material

As a kind of metal oxide semiconductor, 2D h-WO_3_ gas sensing material has been an excellent candidate material for gas sensors due to its advantages of easy preparation, stable crystal structure, high specific surface area, and abundant oxygen vacancies.

### Easy Preparation

Table 1 shows some typical preparation methods of 2D h-WO_3_. Among them, the hydrothermal method is the most widely used. According to this method (Kitagawa et al., 2009; Szilágyi et al., 2010; Ji et al., 2019b), (NH_4_)_10_W_12_O_41_∙5H_2_O is firstly put into a high-pressure cauldron as the raw material. Then, under high temperature and high pressure, (NH_4_)_10_W_12_O_41_∙5H_2_O recrystallizes to obtain precipitates (h-WO_3_ crystals). Finally, the precipitates are removed and washed several times with deionized water to obtain the final product. Compared with vapor/liquid phase deposition methods, the hydrothermal method is simple and economical, and can prepare nanomaterials with high purity, good chemical uniformity and high dispersion. 2D h-WO_3_ is classified as the surface-controlled gas sensor by a gas sensing mechanism.

**TABLE 1:** The preparation methods and types of 2D h-WO_3_.

Structure	Materials	Method	Gas	Type
2D h-WO_3_	Nanosheet	Hydrothermal method	NH_3_ ^a^	Surface-controlled gas sensor
	Nanosheet	Hydrothermal method	H_2_S^b^	
	Film	Hydrothermal method	NO_2_ ^c^	
	Film	Sol-gel polymerization	H_2_ ^d^	
	Film	Acidic precipitation	NH_3_ ^e^	

a Ji et al.(2019b).

b Szilágyi et al.(201).

c Kitagawa et al.(2009).

d Zhang et al.(2019).

e Balázsi et al.(2008).

### Stable Crystal Structure

**FIGURE 2:**
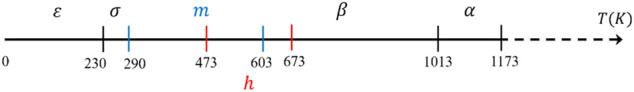
Stability temperature domains of the different polymorphs of WO_3_ (Gerand et al., 1979; Roussel et al., 2000).

The phases of WO_3_ can transform when it is annealed under different conditions. However, it does not simply form new nanostructures, but the original WO_6_ octahedron distorts and twists to a certain extent and thus can form different crystal phases. The phase transition with temperature of WO_3_ is shown in Figure 2 (Salje et al., 1997; Vogt et al., 1999; Roussel et al., 2000), which is monoclinic II (ε-WO_3_ < −43°C) → triclinic (−43°C < σ-WO_3_ < 17°C) → monoclinic I (17°C < m-WO_3_ < 330°C) → orthorhombic (330°C < β-O_3_ < 740°C) → tetragonal (740°C < α-WO_3_). Meanwhile, Gerand et al. (1979) found that stable hexagonal WO_3_ (h-WO_3_) can be prepared by dehydration method at 200°C–400°C.

Tian et al. (2020) has calculated the gas (O_2_) sensing on hexagonal WO_3_ (001) surface by using the pseudopotentials method based on the density functional theory (DFT). The formation energy (*E*_form_) of the h-WO_3_ (001) monolayer is −5.87 eV, indicating that the h-WO_3_ (001) monolayer is stable. The carrier mobility *μ* calculated from the energy band is 886 cm^2^V^−1^s^−1^ (as shown in Table 2) at *T* = 300 K. The value is higher than that of 2D GeP_3_ (Gerand et al., 1979) and MoS_2_ (Cai et al., 2014) and is about 88 times higher than that of bulk WO_3_ (Yamazoe et al., 2003), which implies that 2D h-WO_3_ may have excellent gas sensing performance.

**TABLE 2:** The carrier mobility *μ* at *T* = 300 K.

Material	*μ* (10^3^ cm^2^ V^−1^ s^−1^)
h-WO_3_ monolayer^a^	0.886
Graphene^b^	15.000
InP_3_ ^c^	1.919
SnP_3_ ^d^	7.150
GeP_3_ ^e^	0.360
MoS_2_ ^f^	0.201
2D MoS_2_ flake^g^	0.600
SnO_2_ bulk^h^	0.160
WO_3_ bulk^h^	0.010

a Sone et al.(2018).

b Novoselov et al.(2004).

c Miao et al.(2017).

d Ghosh et al.(2018).

e Gerand et al.(1979).

f Cai et al.(2014).

g Alsaif et al.(2016).

h Yamazoe et al.(2003).

### High Specific Surface Area

Sun et al. (2015) investigated the high surface area tunnels in 3D h-WO_3_ by low-pressure CO_2_ adsorption isotherms with nonlocal density functional theory fitting (NLDET), transmission electron microscopy (TEM), and thermal gravimetric analysis. They found that h-WO_3_ has a large hexagonal tunnel structure (the diameter is 3.67 Å) and high specific surface area (45.585 m^2^/g). Meanwhile, the large lateral size and ultrathin thickness of 2D materials provide it with ultrahigh specific surface areas and high ratios of exposed surface atoms (Zhang, 2015), significantly improving the gas sensing performance of 2D h-WO_3_.

### Abundant Oxygen Vacancies

The conduction band of 2D WO_3_ mainly consists of W-5d electrons, and the valence band mainly consists of O-2p electrons (Niklasson et al., 2004). Chatten et al. (2005) found that abundant oxygen vacancies are related to the energy gap between O-2p and W-5d orbitals in non-stoichiometric tungsten oxide. Makarov and Trontelj (1996) pointed out that the oxygen vacancies in 2D WO_3_ can affect the conductivity and carrier concentration, and further affect the gas sensing performance of WO_3_. For example, Tian et al. (2020) found that oxygen vacancies provide electrons to O_2_ gas molecules on the WO-terminated h-WO_3_ (001) surface, thus effectively improving the gas sensing performance of h-WO_3_ (001) surface to O_2_.

## Influencing Factors of 2D h-WO_3_ on Gas Sensing Performance

When the gas sensors are exposed to the air, O_2_ molecules are physically or chemically adsorbed on the surface of 2D h-WO_3_. The oxygen will be dissociated and capture the electrons from the conduction bands of 2D h-WO_3_, generating ionized oxygen species (mainly O^−^). This leads to a decrease in the number of electrons on the surface and forming an electron depletion region (EDR), which causes the first change in resistance. When the sensors are exposed to the target gas, the gas molecules are adsorbed on the surface of 2D h-WO_3_. Then, the gas molecules react with pre-absorbed oxygen and change the number of the electrons of ionized oxygen species, increasing the density of carriers in the 2D h-WO_3_. It results in the second change in resistance (Deng et al., 2015; Li et al., 2015; Liu et al., 2016).

### Effect of Microstructure on Gas Sensing Performance of 2D h-WO_3_

**FIGURE 3:**
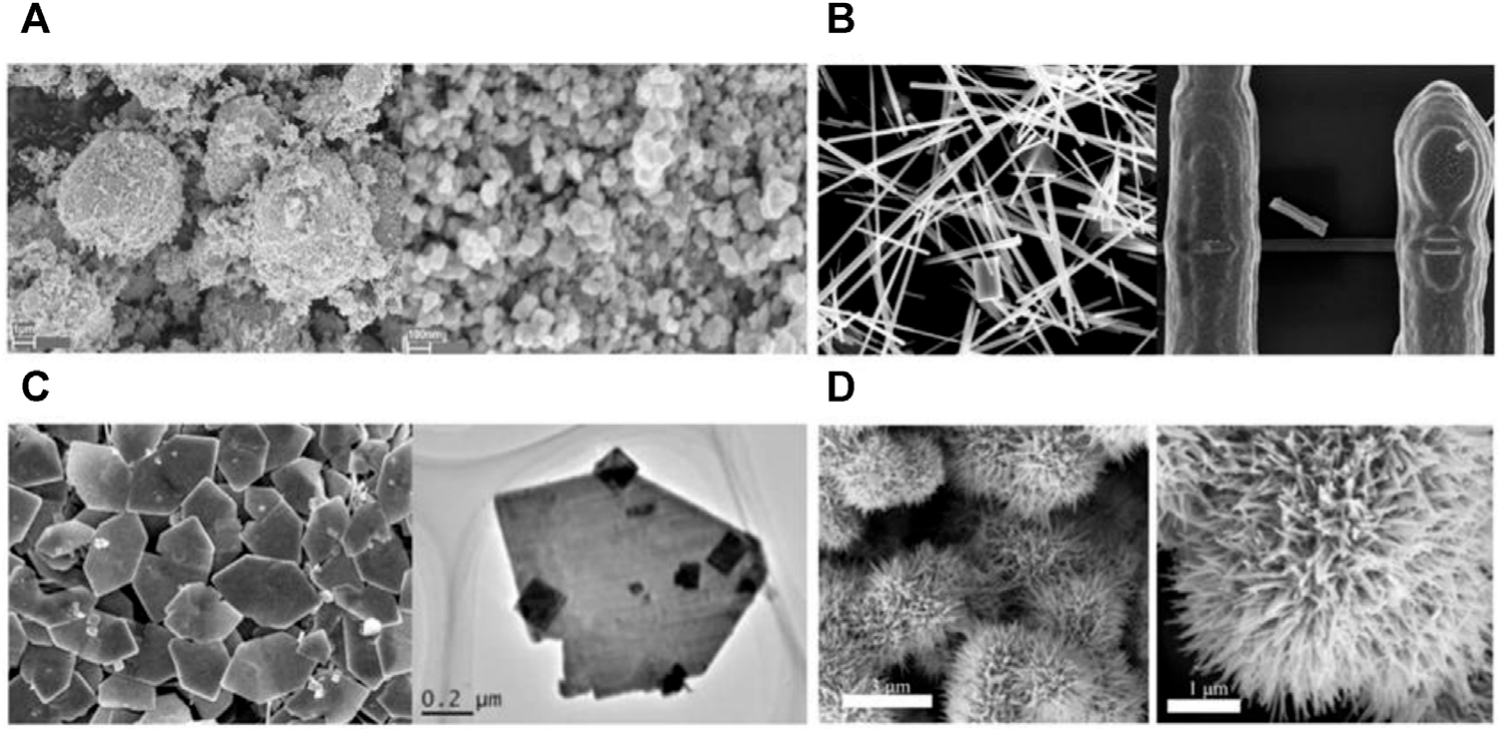
The microstructure of h-WO_3_ nanoparticle, nanowire, film, and nanosphere **(A)** 0D h-WO_3_ nanoparticle (Szilágyi et al., 2010). **(B)** 1D h-WO_3_ nanowire (Liu et al., 2014). **(C)** 2D h-WO_3_ film (Meng et al., 2015). **(D)** 3D h-WO_3_ nanosphere (Zhang et al., 2019).

Figure 3 shows different microstructures of h-WO_3_. It can be seen that h-WO_3_ nanosheets and films can provide more gas molecular absorption sites because of their obvious orientation, small particle size, large specific surface area, and no agglomeration. However, h-WO_3_ nanoparticles, nanowires, and nanospheres have a negative effect on gas transportation and reaction due to serious agglomeration or large particle size. Moreover, we also find from Table 3 that h-WO_3_ nanosheets and films have the highest responsiveness (*R*) and wider detection scope (*S*) to H_2_, NH_3_, H_2_S, and NO_2_, compared with nanowires, nanorods, nanospheres, and nanoparticles. Different h-WO_3_ nanomaterials have exhibited different gas sensing performance due to their different microstructures. Among them, 2D h-WO_3_ nanomaterials show important application prospects in the gas sensing field due to their excellent gas sensing performance.

**TABLE 3:** Relationship between microstructure, particle size, and gas sensitivity of H_2_, NH_3_, H_2_S, and NO_2_ in h-WO_3_ (*S* is the detection scope, *R* is the responsiveness, and *C* is concentration).

Gas	Material	Size/nm	T/°C	S/ppm	R	
					R = R_a_/R_g_	C/ppm
	Film^a^	110–320	450	200	151.9	200
H_2_	Nanoflower^b^	450–600	270	100	2.5–5	100
	Nanosphere^c^	500–2000	250	10–80	0–5	80
	Nanoparticle^d^	50–100	300	10–50	5–5.5	50
NH_3_	Nanorod^e^	30–100	400	50–200	22.5	200
	Nanosheet^f^	50–350	350	50–250	36.3	100
	Nanoparticle^g^	50–100	200	—	—	200
H_2_S	Nanowire^h^	50–500	20	—	—	—
	Nanosheet^i^	—	330	0–40	45.86	40
	Nanoparticle^j^	700–1,000	75	1–10	5.8	10
NO_2_	Film^k^	1,000–2000	200	0.01–0.5	10^4^	0–0.1
	Nanosphere^l^	500–2000	250	10–80	60–65	80

a Sone et al.(2018).

b Zhang et al.(2019).

c Wei et al.(2017).

d Wang et al.(2007).

e Szilágyi et al.(2009).

f Ji et al.(2019b).

g Liu et al.(2014).

h Shi et al.(2016).

_i_ Szilágyi et al.(2010).

j Meng et al.(2015).

k Kitagawa et al.(2009).

l Zhang et al.(2019).

### Effect of Oxygen Vacancy on Gas Sensing Performance of 2D h-WO_3_

In 1964, Kevane (1964) found that oxygen vacancies are easy to form in the preparation of metal oxide semiconductors. Makarov and Trontelj (1996) found that the oxygen vacancies would affect the conductivity, thus further affecting the gas sensing performance of WO_3_. However, the expression of oxygen vacancy on metal oxide semiconductor surfaces is not in agreement (Gillet et al., 2003). Until 2018, Tian et al. (2018) established a theory based on surface oxygen density (*do*) and unitedly expressed the oxygen vacancies on the WO_3_ surface (Table 4). The O-terminated and WO-terminated h-WO_3_ (001) surfaces with and without oxygen vacancy are denoted as O- for O-terminated, Vac O- for defective O-terminated, WO- for WO-terminated, and Vac WO- for defective WO-terminated, respectively. The surface oxygen densities are defined as *d*_*o*_ = 1, 1 > *d*_*o*_ > 0, *d*_*o*_ = 0, 0 > *d*_*o*_ > −1. Based on this, oxygen vacancies of the 2D h-WO_3_ surface can be expressed by surface oxygen density.

**TABLE 4:** The relationship between surface oxygen vacancy and oxygen density of 2D h-WO_3_ (Tian et al., 2018).

2D h-WO_3_(001)	Surface oxygen density *d*_*o*_
O-	1
Vac O-	1 > *d*_*o*_ > 0
WO-	0
Vac WO-	0 > *d*_*o*_ > −1

Recently, Tian et al. (2014) investigated the effect of oxygen vacancy on the gas sensing performance of CO on 2D h-WO_3_ (001) surface by using the first-principles calculations (Table 5). They found that the adsorption energy and charge transfer of CO of the defective O-terminated h-WO_3_ (001) surface decrease by 0.68 eV and 0.002e, respectively, compared with the O-terminated h-WO_3_ (001) surface. For defective WO-terminated, the values of decrease are 0.4 eV and 0.011e, respectively. The result shows that the adsorption and sensing ability of CO on the defective O- and WO-terminated h-WO_3_ (001) surface decreases. The oxygen vacancy inhibits the oxidation reaction of reducing gas CO on the 2D h-WO_3_ (001) surface, which reduces the gas sensing performance of the 2D h-WO_3_.

**TABLE 5:** The adsorption energy and charge transfer of O_2_, CO, H_2_, H_2_S, and CH_4_ on 2D h-WO_3_ (001) surface with oxygen vacancy (*d*_*o*_ is surface oxygen density, *C* is charge transfer, Δ*C* is the variation of charge transfer, ↑ is promotion, ↓ is reduction).

Gas	*d*_*o*_	Configurations	E_ads_/eV	ΔE_ads_/eV	C/e	ΔC/e	Effect
CO^a^	1	OC-O_1c_	2.64	0	0.5	0	—
	1 > *d*_*o*_ > 0	OC-O_1c_	1.96	−0.68	0.498	−0.002	↓
	0	OC-W_5c_	0.97	0	0.14	0	—
	0 > *d*_*o*_ > −1	OC-W_5c_	0.57	−0.4	-0.129	−0.011	↓
H_2_S^b^	1	H_2_S/Pt_4_	2.78	0	0.483	0	—
	1 > *d*_*o*_ > 0	H_2_S/Pt_2_	1.85	−0.93	0.474	−0.009	↓
H_2_ ^c^	1	H_2_-O_1c_-P	2.62	0	0.635	0	—
	1 > *d*_*o*_ > 0	H_2_-Pre-O_1c_	0.60	−2.02	0.621	−0.014	↓
	0	H_2_-O_2c_-P_1_	0.19	0	0.09	0	—
	0 > *d*_*o*_ > −1	H_2_-W_4c_-P	0.16	−0.03	0.065	−0.025	↓
CH_4_ ^d^	1	H_2_CH_2_-O_1c_	0.12	0	0.012	0	—
	1 > *d*_*o*_ > 0	HCH_3_-W_5c_	0.18	−0.06	0.049	+0.037	↓
	0	H_2_CH_2_-W_5c_	0.11	0	0.01	0	—
	0 > *d*_*o*_ > −1	—	−6.15	—	—	—	↓
O_2_ ^e^	1	O_2_-O_1c_-P	0.19	0	0.198	0	—
	1 > *d*_*o*_ > 0	O_2_-W_5c_-P	0.24	+0.05	−0.094	−0.104	↑
	0	O_2_-O_1c_-V	1.65	0	−0.389	0	—
	0 > *d*_*o*_ > −1	O_2_-Vac-V	7.30	+5.65	−0.466	+0.077	↑

a Tian et al. (2014).

b Szilágyi et al.(2010).

c Tian et al.(2017).

d Wu et al.(2019).

e Tian et al.(2020).

Oxygen vacancy also inhibits the gas sensing performance of other reducing gases (H_2_S, CH_4_, H_2_) on the 2D h-WO_3_ (001) surface (Szilágyi et al., 2010; Tian et al., 2017; Wu et al., 2019) (Table 5). However, the inhibitory effect of oxygen vacancy on H_2_S and CH_4_ is unapparent. Although the gas sensing performance of H_2_S is inhibited by oxygen vacancy, the value (1.85 eV) is still large enough for effective adsorption of H_2_S on the surface. The adsorption sensing ability of CH_4_ on the 2D h-WO_3_ (001) surface is weak and the inhibition of oxygen vacancy makes it difficult to spontaneously adsorb on defective WO-terminated h-WO_3_ (001) surface. Moreover, oxygen vacancy has the strongest inhibitory effect on the gas sensing performance of H_2_ on the 2D h-WO_3_ (001) surface. The adsorption energy decreases from 2.62 to 0.16 eV and the charge transfer decreases from 0.635e to 0.065e. The gas adsorption ability of H_2_ on the 2D h-WO_3_ (001) surface greatly reduces with the decrease of surface oxygen density.

More recently, Tian et al. (2020) investigated the effect of oxygen vacancy on the gas sensing performance of O_2_ on the 2D h-WO_3_ (001) surface (Table 5) by the first principles with pseudopotentials method based on the DFT. They found that the adsorption energy of O_2_ of the defective O-terminated h-WO_3_ (001) surface increases by 0.05 eV and the charge transfer decreases by 0.104e compared with the O-terminated h-WO_3_ (001) surface. For the defective WO-terminated surface, the values of increase are 5.65 eV and 0.077e, relatively. The result shows that the adsorption and sensing ability of O_2_ are improved on the defective O- and WO-terminated h-WO_3_ (001) surface. The oxygen vacancy activates the O-O bond of O_2_ and promotes the reduction reaction of oxidizing gas O_2_ on the 2D h-WO_3_ (001) surface, which improves the gas sensing performance of the 2D h-WO_3_.

These results indicate that the effect of oxygen vacancy on gases with different redox properties is different. For reducing gases, the oxygen vacancy inhibits their oxidation reactions on the 2D h-WO_3_ (001) surface and then reduces the gas sensing performance of the reducing gases. On the contrary, for oxidizing gases, the oxygen vacancy promotes the reduction reaction and then improves the gas sensing performance.

### Effect of Doping Modification on Gas Sensing Performance of 2D h-WO_3_

Various methods have been performed to improve the gas sensing performance, to overcome the defects of pure metal oxides such as low sensitivity, low selectivity, and long response time for some gases (Liu et al., 2019). Among them, noble metal doping is one of the most common and effective methods. Due to the high electronic activity of noble metal elements, the activation energy of the reaction can be reduced during the contact reaction between the gas sensing material and the target gas, thus improving the gas sensing performance of the materials (Xu et al., 1990) when they react with target gases. Based on this, noble metals such as Au, Ag, Pd, and Pt are usually doped on WO_3_ films to improve their sensitivity and selectivity to NO_*x*_ (Penza et al., 1998; Chen and Tsang, 2003), H_2_S (Stankova et al., 2004; Hurtado-Aular et al., 2021), CH_3_COCH_3_ (Feng et al., 2021), etc.

Recently, the gas sensing performance of CO adsorption on the 2D h-WO_3_ (001) surface doped with noble metals Cu, Ag, and Au were investigated by using DFT (as shown in Table 6) (Hurtado-Aular et al., 2021). They found that the incorporation of Au and Cu atoms improves the surface activity of the material and the absorptivity of CO on the 2D h-WO_3_ (001) surface. Meanwhile, the doped Au and Cu atoms provide a large number of electrons. The charge transfer increases, which effectively improves the sensing performance of CO on the 2D h-WO_3_ (001) surface.

**TABLE 6:** Adsorption energy and charge transfer of CO and H_2_S on noble metal doped 2D h-WO_3_ (001) surface.

Gas	Surface	E_ads_/eV	Charge transfer/e
CO^a^	Clean	−0.69	+0.08
	Cu	−1.79	+0.02
	Ag	−0.97	+0.04
	Au	−2.06	+0.07

a Hurtado-Aular et al.(2021).

Theoretically, noble metal doping promotes the adsorption and sensing ability of the target gas on 2D h-WO_3_ surface, and then improves the gas sensing performance of 2D h-WO_3_. However, the experimental study on the mechanism of improving the gas sensing performance of noble metal doped h-WO_3_ films is still insufficient.

## Summary and Prospect

The research progress of the gas sensing performance of 2D h-WO_3_ has been reviewed. Firstly, we briefly summarize the characteristics of 2D h-WO_3_ gas sensing materials. Then, the effects of microstructure, oxygen vacancy, and doped metal on the performance of 2D h-WO_3_ gas sensors are mainly discussed. We find that the 2D h-WO_3_ gas sensor has better gas sensing performance than other WO_3_ nanomaterials due to their small particle size and large specific surface area. Moreover, the effect of oxygen vacancy on the gas sensitivity of different oxidation-reducing gases on 2D h-WO_3_ is different. Besides, we also note that noble metal doping can improve the gas sensing performance of 2D h-WO_3_ due to the high electronic activity of noble metals and the reduction of reaction activation energy.

As we all know, 2D h-WO_3_ is an excellent candidate material for metal oxide semiconductor gas sensors, which has vital research significance and wide application prospects in gas sensors. However, there are still some unsolved problems in 2D h-WO_3_ that need to be completely solved, such as the low sensitivity and low selectivity to some gases. To solve the above problems, the possible solutions include the following: (1) Photoactivation method (i.e., activation of reactants by light), which can improve the sensitivity and selectivity effectively. Deng et al. (2012) activated mesoporous WO_3_ sensing material and improved the sensitivity of WO_3_ to HCHO by using visible light irradiation at room temperature. Moreover, Trawka et al. (2016) enhanced the sensitivity and selectivity of WO_3_-based gas sensors greatly by ultraviolet irradiation. (2) Noble metal doping method improves sensitivity and selectivity. Adding precious metal catalysts has become an important method to improve the gas sensing performance of metal oxide semiconductors, because the catalyst has a great influence on the resistance and sensitivity of semiconductor gas sensing materials (Krebs and Grisel, 1993).
